# New UHPLC-QqQ-MS/MS Method for the Rapid and Sensitive Analysis of Ascorbic and Dehydroascorbic Acids in Plant Foods

**DOI:** 10.3390/molecules24081632

**Published:** 2019-04-25

**Authors:** Nieves Baenas, Francisco J. Salar, Raúl Domínguez-Perles, Cristina García-Viguera

**Affiliations:** 1 Institute of Nutritional Medicine, University Medical Center Schleswig-Holstein, Campus Lübeck, Ratzeburger Allee 160, 23538 Lübeck, Germany; nieves.baenas@uni-luebeck.de; 2 Phytochemistry and Healthy Food Lab, Group on Quality, Safety and Bioactivity of Plant Foods, Department of Food Sciences and Technology, CEBAS-CSIC, University Campus of Espinardo, Edif. 25, 30100 Murcia, Spain; fjsalar@cebas.csic.es (F.J.S.); cgviguera@cebas.csic.es (C.G.-V.)

**Keywords:** validation, UHPLC, MS/MS, vitamin C, matrix effect

## Abstract

A new method using ultra high-performance liquid chromatography coupled with triple quadrupole mass spectrometry (UHPLC-QqQ-MS/MS) methodology was developed for the determination of ascorbic acid (AA) and dehydroascorbic acid (DHAA) contents in liquid and solid vegetable samples. The advantages of this method are speed, high sensitivity and practical application. In accordance with these advantages, the present method allows the simultaneous determination of AA and DHAA without previous reduction/derivatization of DHAA and without the use of internal standards in the samples. This is of high interest in routine analysis, providing a simpler sample preparation, as well as enhanced accuracy and robustness. Its validation included selectivity, sensitivity and linearity, precision and accuracy, matrix effect, and recovery. The results showed high selectivity and sensitivity, with calibration curves ranging from 10 to 500 ng mL^−1^ and from 50 to 500 ng mL^−1^ for AA and DHAA, respectively. Appropriate dilutions for each sample are necessary to avoid the matrix effect with accepted recoveries.

## 1. Introduction

Vitamin C is one of the most important water-soluble vitamins for health. It is unable to be synthesized in humans and is involved in many biochemical functions, such as neutralization of free-radicals, the absorption of iron at the gastrointestinal level, and the synthesis and protection from oxidation of collagen, catecholamines, cholesterol, amino acids and some peptide hormones as an essential enzyme cofactor [1]. The Recommended Dietary Allowance (RDA) of vitamin C is 90 and 75 mg per day, on average, for adult men and women, respectively [2], although specific pathophysiological states can modulate the requirements of this vitamin [3]. Subsequently, vitamin C may play a major role in the initiation and progression of diverse chronic and acute diseases related to oxidative damage and inflammation [4]. On the other hand, from a technological point of view, vitamin C could be used as an antioxidant preservative in food and pharmaceutical industries, contributing to protecting manufactured products from spoilage [5].

The methodology for vitamin C analysis should involve the quantification not only of L-ascorbic acid (AA), but also of its oxidation product, the dehydroascorbic acid (DHAA), as this compound exhibits equivalent biological activity to AA [6,7]. Therefore, the evaluation of only AA may lead to an underestimation of the biological value of a given food matrix as a source of this vitamin [8]. Among the different technologies available for the quantification of vitamin C, such as spectrophotometry, titration, or enzymatic methods, the high-performance liquid chromatographic (HPLC) methods are preferred [9], due to their separation capability, high selectivity, and accuracy. Most HPLC methods described so far are based on ultraviolet-visible (UV-Vis), diode array detector or photo diode array (DAD/PDA) detection systems, which identify total AA by transforming DHAA to this compound upon a redox reaction, the so-called “subtraction methods” [10,11,12]. This process requires long reaction times and the use of reducing agents. DHAA can also be analyzed after pre-column derivatization with *O*-Phenylenediamine (OPDA) to form its highly fluorescent quinoxaline derivative, allowing its detection by fluorescence (FLD) [13,14], however, this multi-step method is very time-consuming. Additionally, OPDA is considered by the European Union (EU) REGULATION (EC) No 1272/2008 as a health and environmental hazard with high toxicity, which suggests a strong interest in reducing its use, for instance by developing new techniques that do not need it.

Ultra-high-performance liquid chromatography (UHPLC) is the crucial advance of chromatographic separation techniques, showing higher efficiency and resolution, shorter time analysis, lower solvent consumption, and increased sensitivity relative to HPLC methodologies [10,12,15]. Due to this, UHPLC-DAD methods for vitamin C quantification have been recently developed [16,17]. Nevertheless, a previous step of reduction/derivatization of DHAA continues to be necessary for total vitamin C evaluation. 

In order to avoid this multi-step method, the use of mass spectrometer technology (MS/MS) coupled with the UHPLC could allow the simultaneous determination of AA and DHAA, without carrying out reduction or derivatization reactions. Regarding this technology, Fenoll et al., (2011) reported a method based on HPLC separation system coupled to electrospray ionization (ESI) and MS/MS detection, which allows the simultaneous analysis of AA and DHAA [15]. However, in this method, internal standards were used in every sample to allow the detection of the analytes. It is perhaps due to the high matrix effect found in the samples, that constitutes a limiting factor in HPLC methods due to the lower sensitivity of this technique compared with UHPLC methods, especially those operated in multiple reaction monitoring (MRM) mode [18].

In accordance to these antecedents, the development and validation of a new method that provides reliable analytical data and practical application is essential. In this sense, the present work pursued the development of a new and improved UHPLC-MS/MS-based method regarding rapidness and simple sample preparation, which allows the simultaneous determination of AA and DHAA, without previous reduction/derivatization of DHAA, as well as the analysis of samples without the use of internal standards, revealing high sensitivity and accuracy. In addition to the chromatographic settings, and with the purpose of studying and avoiding the limitations associated to the determination of AA and DHAA in different food matrices, these compounds were evaluated in three different foods rich in vitamin C: fresh orange juice (liquid matrix) and freeze-dried fresh orange juice (same sample but processed to provide a solid matrix), and freeze-dried broccoli florets (different source but also a solid matrix).

## 2. Results and Discussion

The validation of this method was performed according to the International Guidelines on Validation of Analytical Procedures (ICH) [19] and the FDA Guidance for Bioanalytical Method Validation [20]. Chromatographic conditions were also adjusted to reduce the analysis time, minimize sample preparation, and eliminate the matrix effect of the samples, compared with previous HPLC and UHPLC methods reported in the scientific literature. Fresh orange juice and freeze-dried fresh orange juice were used as a liquid matrix sample and as a solid matrix sample, respectively, representing samples with high amounts of AA. In addition, freeze-dried broccoli florets were used as vegetables with high content in DHAA.

### 2.1. Qualitative and Quantitative MRM Transitions

**Figure 1:**
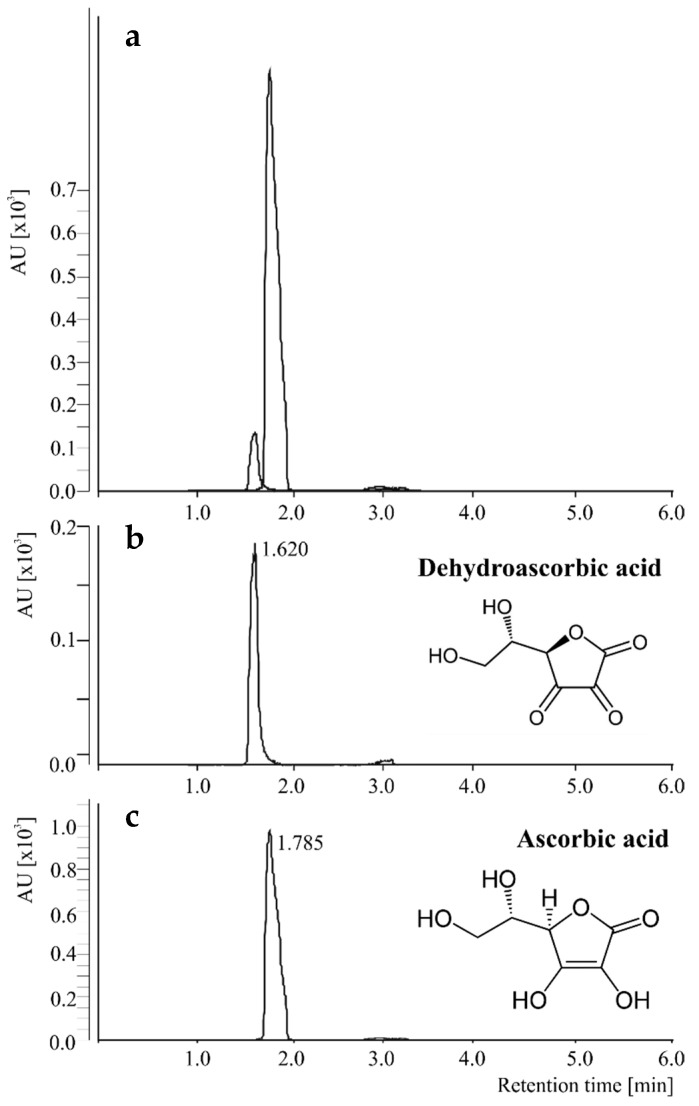
Representative overlap and individual spectra of dehydroascorbic (**a** and **b**, repectively), and ascorbic (**a** and **c**, respectively) acids obtained by UHPLC-ESI-QqQ-MS/MS working in the multiple reaction monitoring mode.

In the present method, the negative ESI mode was used to analyze AA and DHAA. The mass spectra analysis was carefully optimized including the selection of the precursor ion and the most abundant fragment ions detected in a second stage of mass spectrometry. Thus, two MRM transitions, both with precursor ion and product ion, were selected for each compound with quantification and confirmation purposes. The selectivity of the method was ensured, providing transitions of AA and DHAA that avoid interferences with other compounds present in the food matrices according to the ICH [19] (Figure 1). The retention times for the analytes were 1.785 and 1.620 min, for AA and DHAA, respectively, further confirmed with the selectivity of the method. The mass spectrometry parameters collision energy (CE) and fragmentor voltage (V) were also optimized according to the maximum intensities of the product ions monitored (Table 1).

### 2.2. Calibration Curves and Sensitivity

Preparation of the calibration curves was carried out daily for AA and DHAA dissolved in EDTA 0.05% (*w*/*v*) as described by Fenoll et al. (2011) [15]. The sensitivity of the analytical method for the detection of both compounds, defined as the lowest analyte concentration that can be measured with acceptable accuracy and precision, as well as the capability of the technical procedure to discriminate differences in their concentrations, were achieved by the evaluation of the limit of detection (LOD). This is defined as the lowest amount of analyte in the sample, which can be detected but not quantified, and the limit of quantification (LOQ), defined as the lowest concentration which is sensitive to be determined quantitatively [19].

The LOD and the LOQ values of the present method were determined at 6.25 and 10 ng mL^−1^, respectively, for AA and 12.5 and 50 ng mL^−1^, respectively, for DHAA. These concentrations were lower than those reported by Fenoll et al. (2011) [15] for AA and DHAA evaluation by HPLC. Additionally, the LOD and LOQ for AA determination were lower than those established by other UHPLC methods, where the content of DHAA were determined indirectly by its reduction to AA [17,18]. Therefore, the present method enhanced the sensitivity to assess food matrices on their content of AA and DHAA compared to previous methodologies.

On the other hand, the linear relationship was defined using the ion chromatography peak response and the concentration of the relevant ion (the calibration graphs were constructed as plotting peak area vs. concentration). The linearity of the standards was evaluated in the range 10–500 ng mL^−1^ and 50–500 ng mL^−1^ for AA and DHAA, respectively, by linear regression equations of the type y = ax + b, and revealed significant correlation coefficients higher than 0.994 for each separate analyte (Table 2). The standard curves were checked by injecting the target analytes separately or both together in a solution, with identical satisfactory results. The evaluation of higher concentrations of both AA and DHAA resulted in a lack of linearity.

### 2.3. Precision and Accuracy

The precision of the method was determined by sequential analysis of the standards and expressed as coefficient of variation (CV%). The intra- and inter-day CV% and the percentages of deviation or accuracy (Table 3) were calculated by analyzing three different concentration levels of standard solutions (80, 200, and 400 ng mL^−1^) prepared daily, within the linearity range of the calibration curve. The intra-day precision of the assay was determined by the repetition of the standard solutions analysis three times (in triplicate each time) within the same day. Regarding the inter-day precision, it was assessed by the analysis of the three concentrations of the standards in three separate days, analyzing three replicates each day.

The results obtained for the CV% (precision) ranged from 2.5 to 13.7% and from 6.3 to 12.2%, for the intra-day and inter-day determinations, respectively. The accuracies obtained varied between 86.4 and 114.8% and between 86.7 and 113.5%, for the intra-day and inter-day, respectively (Table 3). According to the previous results, the intra- and inter-day precisions (lower than 15.0%) and the results on accuracy (within the range of 80.0–120.0%), were considered satisfactory in the present method, being in agreement with the acceptable ranges established by the International Conference of Harmonization [19].

### 2.4. Sample Preparation, Matrix Effect, Sensitivity and Recovery

The matrix effect is considered as the influence of other molecules present in the sample that could cause interferences on the detection and quantification of the target analytes, resulting in over- and underestimated results. Following previously developed methods for AA and DHAA quantification [15,18], several dilutions of the extracted samples (fresh orange juice, freeze-dried orange juice and freeze-dried broccoli florets) were performed, ranging from 1:10 to 1:20. The results obtained revealed clearly defined peaks in the chromatograms, indicating selectivity of the method for both analytes. Nevertheless, no differences in the areas were found due to a strong matrix effect. Subsequently, higher dilutions of the samples were prepared and analyzed, from 1:100 to 1:1000, in order to study the sensitivity of the present UHLPC-MS/MS system.

The results obtained noticed that sample dilution is essential to avoid the food matrix effect, meaning that higher dilutions of the samples (reducing possible interfering compounds) have lower impact on the ionization of the target analytes. In this sense, different dilutions were found appropriate for each food matrix, showing samples of fresh orange juice, freeze-dried orange juice and freeze-dried broccoli, required optimal dilutions of 1:400, 1:200 and 1:100, respectively. 

The requirement of higher dilutions in the present method compared to previous published works are due to an elevated sensitivity of the UHPLC-QqQ-MS/MS analytical instrumentation. Thus, the use of internal standards for AA and DHAA quantification required for conventional HPLC methods (featured by lower sensitivities) were not necessary, providing the advantage of less time-consuming sample preparation [15]. Similar dilutions of the samples were performed by Klimczak & Gliszczyńska-Świgło (2005) using a UHPLC method [18], however, in this work the analysis of DHAA was determined after its reduction to AA.

The evaluation of the consistency, repeatability, and reproducibility of the method by calculating relative standard deviations (RSD%) (precision), matrix effects (ME%), and absolutely recoveries (RE%) of AA and DHAA determinations (Table 4) were developed by adding the internal standards to the samples (both AA and DHAA), at three different concentrations (low level, 80 ng mL^−1^; medium level, 200 ng mL^−1^, and high level, 400 ng mL^−1^) and analyzing the samples in triplicate (*n* = 3). Results of RSD were less than 15% according to the International Conference on Harmonization [19] and the FDA Guidelines for Bioanalytical Method Validation [20]. The ratio “(standards in the samples/standards in water)*100” was performed for matrix effect (ME%) calculations and the ratio “(standard spiked concentrations/standards in the samples)*100” was used for calculation of the absolute recovery parameter (RE%). Both parameters were in the range of 85–120% of the error for the accuracy accepted to guarantee the consistency of analytical determinations [19,21].

Fresh and freeze-dried orange juices represented samples with high amounts of AA, and freeze-dried broccoli florets were chosen as a food matrix with a high content of DHAA. The contents of AA and DHAA in the different vegetables matrices were as follows: fresh orange juice contained 33.69 ± 2.49 mg 100 mL^−1^ of AA; freeze-dried orange juice presented 2.86 ± 0.20 mg g^−1^ dry weight (D.W.) of AA, and freeze-dried broccoli florets presented 9.94 ± 0.98 mg g^−1^ D.W of DHAA. In both fresh and freeze-dried fresh orange juice samples DHAA was not found, according to previous works showing concentrations of 0–0.3% DHAA in fresh juices, as this oxidation product of AA is generally formed during storage [10,18]. In the case of broccoli florets, results showed only the presence of DHAA, which could be due to the rapid oxidation of AA to DHAA during storage, as it has been reported before, only after 4-6 days following harvest [22,23]. Another reason could be that high concentrations of AA were found when broccoli was analyzed fresh [17,24], while processing generally affects AA, showing those samples with higher amounts of DHAA, as shown after freeze-drying [25] or crushing [26].

When determining the operability of the method by its application to the quantification of the content of vitamin C of the drug Redoxon®, different dilutions of the sample were performed, in order to identify acceptable value of areas, while avoiding the matrix effect. In accordance to the Bioanalytical Guidelines of EMA [22] and FDA [20], when a target compound is presented in concentrations exceeding the range of quantification (below or above the LOQ according to the standard curve), the sample can be accurately measured by the dilution linearity assay, or the standard curve could be extended and revalidated. In the present method, the standard curve could not to be extended because of the lack of linearity; therefore, the dilution linearity assay was performed to bring the concentration of the analytes into the validated range for analysis. The selected dilution rate was 1:10,000 for Redoxon®, containing 1165.55 ± 43 mg of AA per tablet, confirming an appropriate CV% which was < 5% among dilutions.

## 3. Materials and Methods 

### 3.1. Chemicals

All reagents and standards were of analytical grade. All solutions were prepared with ultrapure water from a Milli-Q Advantage A10 ultrapure water purification system (Millipore, Kankakee, IL, USA). The target compounds, AA and DHAA, were obtained from Acros Oganics (New Jersey, NJ, USA) and Sigma-Aldrich (St. Louis, MO, USA), respectively. Methanol and acetonitrile of MS grade, and ethylenediaminetetraacetic acid disodium salt 2-hydrate (EDTA) were acquired from Panreac (Barcelona, Spain). Formic acid was purchased from J.T. Baker (Deventer, The Netherlands).

### 3.2. Sample Preparation

For the development of the method, oranges and broccoli florets were purchased from a local market. Fresh orange juice was prepared daily for the analysis as a liquid sample. Freshly prepared orange juice and broccoli florets were rapidly frozen and freeze-dried for their analysis as a solid matrix. For sample processing, the methodology described by Fenoll et al. (2011) [15] was applied with minor modifications. The samples were extracted with EDTA 0.05% (*v/v* or *w/v*) in order to avoid degradation of the target compounds, which remained stable up to 24 h after extraction. The juice or freeze-dried material (3 mL or 3 g, respectively) were mixed with 10 mL of EDTA 0.05% for 2 min using a vortex. Afterwards, samples were centrifuged for 10 min, at 3000 rpm at room temperature. The supernatants were filtered through a Sep-Pack classic cartridge C18 (Waters, Milford, MA, USA) and through 0.22 µm PVDF Millipore filter (Merck Millipore, Carrigtwohill, Ireland) before analysis. Filtered samples were diluted appropriately before UHPLC-ESI-QqQ-MS/MS analysis.

### 3.3. UHPLC-ESI-QqQ-MS/MS Conditions

Ascorbic acid (AA) and DHAA were chromatographically separated using a UHPLC system coupled with a triple quadrupole tandem mass spectrometer model 6460 (Agilent Technologies, Waldbronn, Germany), operating in multiple reaction monitoring (MRM) and negative electrospray ionization (ESI) modes and using a column Pursuit XRs Diphenyl Pursuit USP L11 A6021100X030 (3.0 × 100 mm, 3.0 µm particle size) supplied by Agilent Technologies (Amstelveen, The Netherland), used in combination with a binary gradient system (solvent A: 0.01% formic acid and solvent B: 100% acetonitrile). The flow rate and injection volume were 0.4 mL min^−1^ and 20 µL, respectively. Both AA and DHAA were separated chromatographically upon the optimized gradient of 5.5 min, starting with 0.05% B for 2 min, reaching 90% B at 2.5 min, maintaining 90% B for 1 min, reaching 0.05% B at 4 min and stabilizing the column for 1.5 min. The MS parameters at the optimized conditions were gas flow 8 L min^−1^; nebulizer 35 psi; capillary voltage 3000 V; nozzle voltage 1000 V; sheath gas temperature 350 °C, and jet stream gas flow 11 L min^−1^. Data acquisition and processing were performed by using MassHunter software version B.04.00 (Agilent Technologies, Walbronn, Germany).

### 3.4. Method Validation Parameters

The validation of an analytical method includes studies of selectivity, sensitivity, linearity, precision and accuracy, and assessment of matrix effects and sample recoveries, in accordance to the International Conference on Harmonization [19] and the FDA Guidelines for Bioanalytical Method Validation [20]. The selectivity test was performed by the analysis of AA and DHAA in the different plant foods included in the present work, providing the mass spectra exclusively for the presence of both AA and DHAA analytes without interferences with other compounds. The sensitivity of the analytical method is defined as the lowest analytes concentration that can be measured with acceptable accuracy and precision. This evaluation was performed as the LOD and the LOQ, previously defined in the results section, and were established at a signal to noise (S/N) level of 3/1 and 10/1, respectively, in accordance with the International conference on Harmonization [19]. The linearity is the ability of an analytical method to provide a directly proportional response to the concentration of the analytes in the sample within a given interval. The correlation coefficient of the response function or calibration line is generally accepted to be greater than or equal to 0.970.

The precision, expressed as coefficient of variation (CV%), and accuracy (% deviation) of the analytical method should be measured for intra- and inter- day determination by the evaluation of a minimum of three replicates at three different concentrations of the analyte (in the range of the expected sample concentrations). Results of precision were calculated as “CV% = (standard deviation/mean)*100”. The accuracy was determined as the percentage deviation ratio of the measured concentration to the nominal concentration added (“measured concentration/nominal concentration*100”). A CV% equal or lower than 15% and accuracy within the range 80–120% was considered satisfactory [19,20].

The matrix effect is the influence of all other components of the sample in the quantification of the target compounds in the analytical method. This parameter was determined by adding to the samples three known amounts of the standards (low, medium, and high concentration of AA and DHAA within the calibration line). The matrix effect was evaluated by the percentage change in the signal caused by the matrix components compared to the standards diluted in analytical solvents “ME% = (standards in the samples/standards in water)*100”. The absolute recovery (RE%) may be affected by several factors, including chromatography, ionization at the source, selectivity of the SPE cartridges, and sample matrix. This parameter was evaluated as the percentage of the standards recovered after samples processing compared to the standards added in the samples “RE% = (standards added concentration/standards in the samples)*100” [20].

Finally, in order to assess the proper quantification by the developed method, the pharmaceutical product Redoxon^®^, containing 1000 mg of ascorbic acid per tablet (with an error rate of 8%) was used.

## 4. Conclusions

This new UHPLC-QqQ-MS/MS method showed improvements in speed, sensitivity and practical application, and involved a short time (5.5 min) routine for qualitative and quantitative analysis of both AA and DHAA, in liquid and solid vegetables, with simple sample preparation and robust results, for potential use for pharmaceutical and food analysis. The appropriate dilution of the sample is required for minimizing the food matrix impact on the sensitivity of the method and should be revalidated for the particular type of food matrix in order to achieve the optimal performance according with good analytical practices. 

## Figures and Tables

**Table 1:** Optimal multiple reaction monitoring (MRM) transitions and parameters for quantification of ascorbic and dehydroascorbic acids (AA and DHAA, respectively) by UHPLC-QqQ-MS/MS working in negative ionization mode.

Compound	Retention Time (min)	Quantification Transition ^1^ (*m/z*)	Qualifier Transition (*m/z*)	Collision Energy (eV)	Fragmentor (V)
Ascorbic acid	1.785	175 > 115	175 > 71	5	90
Dehydroascorbic acid	1.620	173 > 143	173 > 113	5	60

^1^ Preferential MRM transitions for quantification of AA and DHAA.

**Table 2:** Calibration data, linearity, limit of detection (LOD), and limit of quantification (LOQ) for ascorbic (AA) and dehydroascorbic (DHAA) acids.

Parameters	AA	DHAA
Range of concentrations for standard curves (ng mL^−1^)	10.00–500.00	50.00–500.00
Calibration curve	y = 67817 x + 766.99	y = 3031.2 x + 440.71
Coefficient of regression (R^2^)	0.9952	0.9936
LOD (ng mL^−1^)	6.25	12.50
LOQ (ng mL^−1^)	10.00	50.00

**Table 3:** Intra-and Inter-day accuracies (%) and precisions (coefficient of variation (CV%)).

Analyte	Intra-Day Accuracy/Precision (CV)			Inter-Day Accuracy/Precision (CV)		
	80 ng mL^−1^	200 ng mL^−1^	400 ng mL^−1^	80 ng mL^−1^	200 ng mL^−1^	400 ng mL^−1^
Ascorbic acid	86.4/10.6	104.2/9.2	98.7/3.1	87.6/12.2	99.2/8.0	90.6/8.3
Dehydroascorbic acid	90.7/13.7	114.8/8.6	95.8/2.5	86.7/10.6	113.5/6.3	98.7/9.9

All values in this table are presented as %.

**Table 4:** Mean percentage of the relative standard deviations or precision (RSD%), matrix effect (ME%) and absolute recoveries (RE%) of ascorbic (AA) and dehydroascorbic (DHAA) acids.

Food Matrix	Nominal Added Concentration (ng mL^−1^)	AA					DHAA				
		Measured	SD	RSD (%)	ME (%)	RE (%)	Measured	SD	RSD (%)	ME (%)	RE (%)
Orange Juice	400	621.42	72.88	13.7	118.6	101.6	464.80	6.79	1.5	118.3	116.8
	200	397.30	41.36	13.3	99.9	104.8	225.13	17.18	7.6	98.0	112.6
	80	267.64	21.98	10.4	107.0	91.4	77.75	8.84	11.4	111.0	97.2
	0	194.53	14.35	7.4	-	-	-	-	-	-	-
Lyophilized Orange Juice	400	574.19	28.66	3.9	103.7	90.0	455.67	30.89	6.8	115.4	113.9
	200	430.11	33.35	8.7	95.2	108.0	180.39	4.90	2.7	85.0	90.2
	80	299.60	25.26	12.4	120.0	106.8	71.02	9.57	13.5	100.8	88.8
	0	214.15	14.64	6.8	-	-	-	-	-	-	-
Lyophilized Broccoli Florets	400	403.42	42.42	10.5	117.8	100.9	3455.24	306.67	2.7	120.1	118.6
	200	174.58	16.28	9.3	86.0	87.3	3216.86	307.63	5.7	102.7	117.9
	80	74.23	5.11	6.9	86.0	92.8	3060.30	302.44	10.4	113.12	99.1
	0	-	-	-	-	-	2981.05	294.24	9.9	-	-

Values shown are means ± standard deviations of three repetitions (*n* = 3). (-) means compound not found.
